# Mixed-reality-based remote endoscopic retrograde cholangiopancreatography education using a three-dimensional anatomical model

**DOI:** 10.1055/a-2871-8031

**Published:** 2026-05-22

**Authors:** Yuki Tanisaka, Shomei Ryozawa, Takao Itoi, Masafumi Mizuide, Akashi Fujita, Ryuichi Watanabe, Maki Sugimoto

**Affiliations:** 1 Department of Gastroenterology 183786 Saitama Medical University International Medical CenterHidakaJapan; 2 Department of Gastroenterology and Hepatology 13112 Tokyo Medical UniversityTokyoJapan; 3 Innovation LabTeikyo University Okinaga Research instituteTokyoJapan

**Fig. 1:**
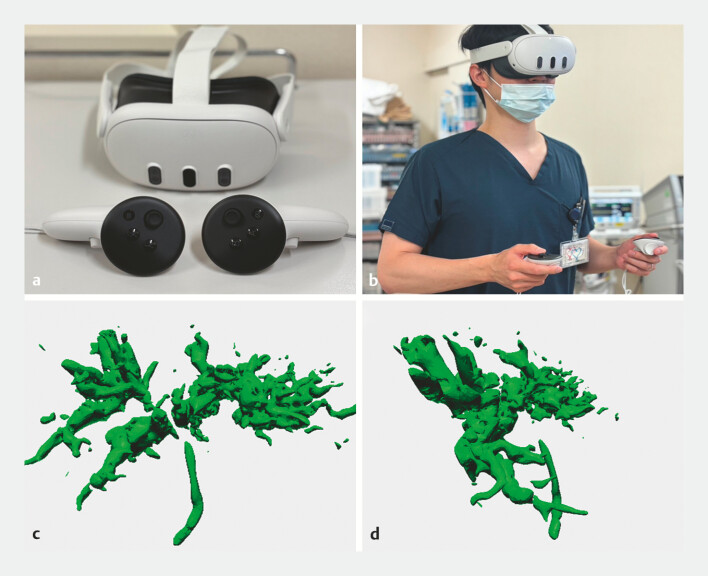
A mixed reality-based three-dimensional (3D) anatomical model. **a** A dedicated head-mounted display and hand controllers. **b** A doctor utilizing a head-mounted display and using hand controllers. **c** A computer-generated 3D anatomical model is seen through the head-mounted display. **d** A 3D anatomical model is rotatable.

Endoscopic retrograde cholangiopancreatography (ERCP)-related procedures for biliary strictures require a thorough understanding of biliary anatomy and accurate identification of the appropriate drainage area. This is particularly challenging in remote areas where experienced ERCP practitioners are not available. Mixed reality (MR) enables the real-time integration of physical and virtual environments, providing an immersive and interactive experience 1 . Using a head-mounted display and controllers (Meta Quest 3, Meta, USA), physicians can visualize and manipulate three-dimensional (3D) anatomical models, which have been reported as useful in pancreatobiliary endoscopy ( Fig. 1 2 3 4 5 ). We report a case of hilar bile duct cancer in which MR-based remote education using a 3D anatomical model was feasible for physicians in a remote area.

**Fig. 2:**
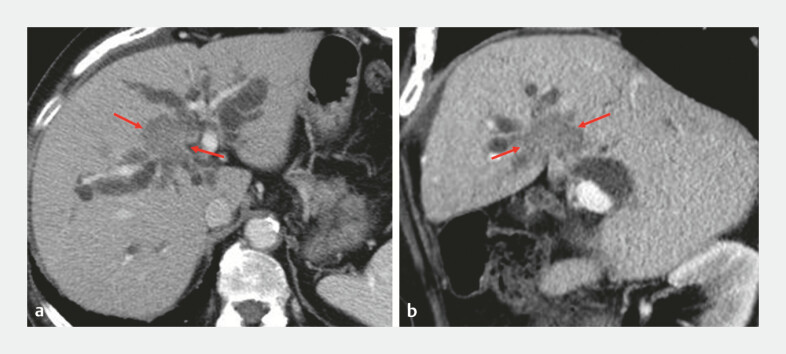
Computed tomography revealing the biliary stricture in the hilar bile duct (a red arrow).

**Fig. 3:**
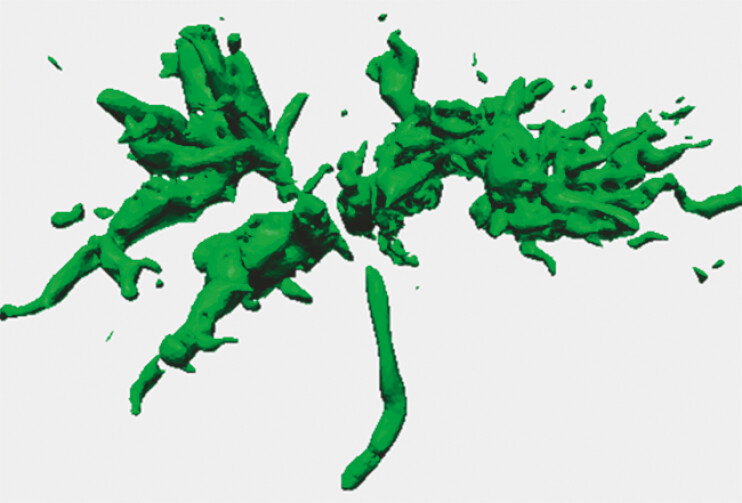
Computed tomography imaging is converted into a three-dimensional (3D) anatomical model.

**Fig. 4:**
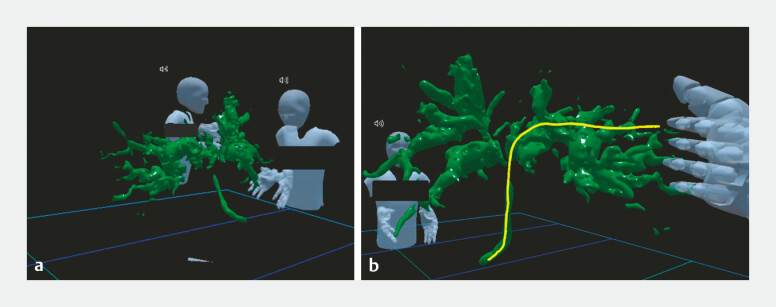
Mixed reality-based remote education using a three-dimensional (3D) anatomical model. **a** Both the local physicians and our team entered a virtual environment as avatars, where remote education was conducted. **b** Since both parties could manipulate the 3D model, the local physicians were able to learn the biliary anatomy and procedural strategy in an immersive manner.

**Fig. 5:**
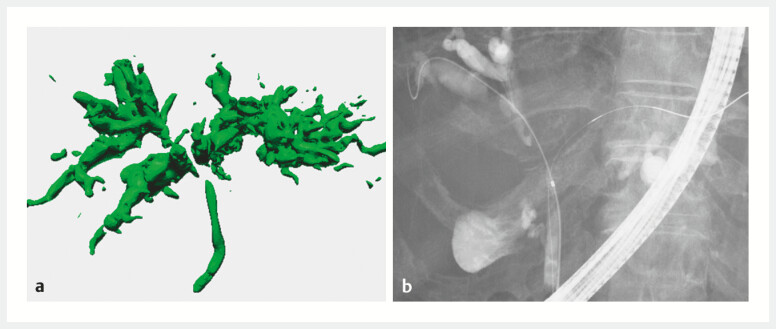
A three-dimensional (3D) anatomical model and cholangiography findings are comparable, making it help their understanding of the procedure.

An 83-year-old man with obstructive jaundice due to hilar bile duct cancer was referred to a rural hospital ( Fig. 2 ). As no experienced ERCP practitioner was available on site, the local physicians consulted our tertiary referral center prior to biliary drainage ( Video 1 ). A 3D anatomical model of the bile duct was created from computed tomography using SYNAPSE VINCENT (Fujifilm Medical Co., Ltd, Japan) and Holoeyes MD software (Holoeyes Inc., Japan; Fig. 3 ). Subsequently, both the local physicians and our team entered a virtual environment as avatars, where remote education was conducted. Since both parties could manipulate the 3D model, the local physicians were able to learn the biliary anatomy and procedural strategy in an immersive manner ( Fig. 4 ). During the procedure, the same model was referenced alongside the real-time fluoroscopic images to help their procedure ( Fig. 5 ). Finally, biliary drainage was successful.

Mixed reality-based remote education for endoscopic retrograde cholangiopancreatography training using a three-dimensional anatomical model in a remote area.Video 1

This case highlights the feasibility of MR-based remote education using a 3D anatomical model for physicians in settings where no experienced ERCP practitioner is available on site, and it may help reduce disparities in ERCP expertise between rural hospitals and tertiary referral centers.

Endoscopy_UCTN_Code_TTT_1AR_2AB
